# High-Entropy Perovskites Pr_1−*x*_Sr_*x*_(Cr,Mn,Fe,Co,Ni)O_3−*δ*_ (*x* = 0–0.5): Synthesis and Oxygen Permeation Properties

**DOI:** 10.3390/membranes12111123

**Published:** 2022-11-09

**Authors:** Zhijun Zhao, Lena Rehder, Frank Steinbach, Armin Feldhoff

**Affiliations:** Institute of Physical Chemistry and Electrochemistry, Leibniz University Hannover, Callinstr. 3A, 30167 Hannover, Germany

**Keywords:** high-entropy oxide, perovskite, mixed ionic–electronic conducting membrane, oxygen separation, sol–gel synthesis

## Abstract

High-entropy perovskite oxides have already been studied in various fields owing to their high-entropy-induced properties. Partial substitution of an element by a lower valence element usually improves the oxygen permeability of perovskite oxides, but high substitution amounts may lead to structural instability. In this work, pure high-entropy perovskites Pr_1−x_Sr_*x*_(Cr,Mn,Fe,Co,Ni)O_3−δ_ with high amounts Sr up to x=0.5 were synthesized via a sol–gel method. Several characterization methods prove that the solubility of Sr increases with higher temperatures of the heating treatment. The ceramic with x=0.5 shows a transition from semi-conductive to metallic behavior when the temperature reaches 873 K. Its oxygen flux is comparable to the low-entropy counterpart La_0.6_Sr_0.4_Co_0.5_Fe_0.5_O_3−δ_. A stable run of ca. 46.2 h was documented for oxygen permeation under an air/CO_2_ gradient.

## 1. Introduction

Since the discovery of single-phase alloys with five equiatomic components in 2004 [1], high-entropy materials have been attracting increasing research interest due to their high-entropy-induced properties, e.g., the tendency to form single phases and great tolerance to lattice distortion [2,3,4,5]. The extension of high-entropy materials is, therefore, continuously expanded. To date, many new systems have been developed, such as high-entropy oxides [6,7,8,9], high-entropy carbides [10,11], and high-entropy metal–organic frameworks [12]. As for perovskites with the general formula ABO_3_, both the M_IIA_(TM)O_3_ family and the RE(TM)O_3_ family can be crystallized to single phase [2,8,9,13,14,15,16], where M_IIA_ and RE are metals of group IIA and rare earth metals on the A-site, and TM stands for transition metals on the B-site.

The effects of high entropy on the performance of perovskites can be summarized in three points. First, due to the probable stabilization effect of high-entropy, perovskites can have enhanced temperature and chemical stability. As an electrode material for solid oxide fuel cells, (La,Nd,Sm,Ca,Sr)MnO_3_ is stable at 1473 K for at least 100 h and has a higher chemical compatibility with the electrolyte 8YSZ (8 mol% Y_2_O_3_ stabilized ZrO_2_) compared to the low-entropy relatives La_1−x_Sr_*x*_MnO_3−δ_ [13]. Second, chemical disorder and lattice distortion can be induced by high entropy, resulting in extra phonon disorder and thus leading to low thermal conductivity and better thermoelectric performance [14,17]. Third, more elements can be packed into pure perovskites, rendering the synergetic effect of cations and improving the catalytic activities of La_0.6_Sr_0.4_(Co,Fe,Mn,Ni,Mg)O_3_ in oxygen evolution reactions [15], Pb(Ni,W,Mn,Nb,Zr,Ti)O_3_ in oxygen reduction reactions [18], and La(Co,Fe,Mn,Ni,Mg)O_3_ in CO oxidation reactions [19].

Although high-entropy perovskites have already been studied in various fields such as proton conducting materials [16,20], electrode materials in solid oxide fuel cells [13,21], thermoelectric materials [14,17], and catalysts [15,18,19], publications about mixed ionic–electronic conductors (MIECs) is rather rare. Wang et al. [22] have found that the A-site and B-site co-doped Ba_0.5_Sr_0.5_Co_0.8_Fe_0.2_O_3−δ_ (BSCF) ceramic Ca_0.1_La_0.02_Gd_0.02_Bi_0.02_Ba_0.42_Sr_0.42_Co_0.736_Fe_0.184_Zr_0.02_Ni_0.02_Cu_0.02_Al_0.02_O_3−δ_ exhibits improved stability of the perovskite structure and the oxygen permeation in the intermediate temperature range due to a stabilization effect caused by its increased mixed entropy. The oxygen permeation of the other type of high-entropy perovskite RE(TM)O_3_, however, is not reported despite its corresponding low-entropy relatives having been extensively studied as oxygen-transporting materials [23,24,25].

The partial substitution of A-site element by group IIA metals (e.g., Sr) is a commonly used strategy to boost the oxygen permeability in the field of oxygen-transporting materials, since it introduces additional ionic charge carriers (i.e., mobile oxygen vacancies) upon high substitution amount [21,26,27]. In addition, the electrical conductivity can also be enhanced by partial substitution due to the $3d_{\mathrm{TM}}$-$2p_{O}$ orbital overlapping and the change of oxidation state of the TM elements on B-site [21,26,27,28]. However, introducing those elements usually escalates the lattice distortion and leads to structural instability. Dąbrowa et al. [21,29] have found that the solubility of strontium is limited in RE(Cr,Mn,Fe,Co,Ni)O_3−δ_, being 0.3 for RE = La and 0.1 for RE = Pr. Secondary phases, e.g., Sr(CO_3_)_2_ or SrCrO_4_, are found when the limit is exceeded. As a comparison, common Sr-doped perovskite oxides, e.g., Pr_1−x_Sr_*x*_(Co_0.5_Fe_0.5_)O_3−δ_, can remain single phase even if x=0.4 [26,30,31,32,33,34]. This is somehow inconsistent with the high-entropy stabilization effect, which is believed that high mixed entropy $\Delta S_{\mathrm{mix}}$ leads to a negative Gibbs free energy $\Delta G_{\mathrm{mix}}$ when $T\Delta S_{\mathrm{mix}}>\Delta H_{\mathrm{mix}}$ as indicated by the following equations (in ideal solid solutions) [4,9]:

(1) $$\Delta G_{\mathrm{mix}}=\Delta H_{\mathrm{mix}}-T\Delta S_{\mathrm{mix}}$$

(2) $$\Delta S_{\mathrm{mix}}=-R\left[{\left(\sum_{a=1}^{n}x\ln x\right)}_{A-\mathrm{site}\;}+{\left(\sum_{b=1}^{n}y\ln y\right)}_{B-\mathrm{site}\;}+3{\left(\sum_{c=1}^{n}z\ln z\right)}_{O-\mathrm{site}\;}\right]$$

where R is the gas constant, and *x*, *y*, and *z* are the mole fraction of elements on A-, B-, and O-sites, respectively. According to Equation (2), Pr_0.6_Sr_0.4_(Co_0.5_Fe_0.5_)O_3−δ_ has a lower mixed entropy of 1.37R compared to Pr_0.9_Sr_0.1_(Cr,Mn,Fe,Co,Ni)O_3−δ_ and Pr_0.5_Sr_0.5_(Cr,Mn,Fe,Co,Ni)O_3−δ_, where the values are 1.93R and 2.30R, respectively.

In this work, as an example of doped RE(TM)O_3_ family, a group of single-phase high-entropy perovskites Pr_1−x_Sr_*x*_(Cr,Mn,Fe,Co,Ni)O_3−δ_ (x= 0–0.5) is synthesized via a sol–gel method with subsequent heating treatments. The influence of Sr content on the electrical conductivity and the oxygen permeability is presented.

## 2. Materials and Methods

### 2.1. Material Synthesis

The chemical formulae of high-entropy perovskite oxides reported in this work are written as Pr_1−x_Sr_*x*_(Cr,Mn,Fe,Co,Ni)O_3−δ_ (x= 0–0.5) as recommended by the International Union of Pure and Applied Chemistry [35]. The nominal amounts of B-site elements are equal, i.e., all have a stoichiometric number of 0.2. The powders were synthesized by adapting a previously reported sol–gel method [36] using stoichiometric amounts of metal nitrates, ethylene-diamine-tetraacetic acid, and citric acid in a molar ratio of 1:1:2. Reactants were purchased from Alfa Aesar and used without further purification. Except for powders studied in phase analysis (Section 3.1), all powders were calcined at 1423 K for 10 h, following by tableting at 300 MPa for 0.25 h and sintering at 1673 K for 10 h (with natural cooling, see Figure A2).

### 2.2. Structural Characterization

The phase purity and crystal structure of products were investigated by X-ray diffraction (XRD) using a diffractometer (D8 Advance, Bruker AXS GmbH) with Cu-K*α* radiation (40 kV and 40 mA, λ = 0.154 nm) and a step size of 0.01° in the 2θ range from 10° to 85°. Rietveld refinements of XRD patterns were performed on the software TOPAS (Version 6, Bruker AXS GmbH). PrCrO_3_ (Pnma, ICSD 251098) and SrCrO_4_ ($P2_{1}/n$, ICSD 40922) were used as starting structures. The elemental composition and microstructure of membranes were examined by two field-emission scanning electron microscopes (FE-SEM): JEOL JSM-6700F equipped with an energy-dispersive X-ray spectrometer (EDXS, Oxford Instruments INCA-300) and JEOL JSM-7610FPlus with twin EDXS (Bruker XFlash 6|60). Before measurements, the cross-sections of membranes were vibratory-polished by VibroMet (Buehler). The backscattered electron channeling contrast images were captured at an acceleration voltage of 15 kV.

### 2.3. Electrical Conductivity Measurements

The sintered membranes were cut into bars with a conductive area of 2 mm^2^ and a length of 10 mm. The sample was fixed between two platinum plates, which were connected to a sweep/function generator (1 Hz square waveform, Wavetek Model 180) and digital multimeters (KEITHLEY 2100, Keithly Instruments) by platinum wires. The measurement cell was heated to 1223 K in a horizontal tube furnace (Carbolite Gero EVZ 12/450N) and the data were recorded by using the software LabVIEW 2015 (Version 15.0.0) at equilibrium conditions during the cooling process.

### 2.4. Oxygen Permeation Measurements

The oxygen permeabilities of sintered membranes were characterized from 1023 K to 1223 K by a home-made high-temperature permeation cell, which is described in detail elsewhere [32]. Before mounting the samples, membranes were polished to 1 mm thick by using 1200-grit sandpaper and washed with ethanol. The sample was then sealed on an alumina tube with a commercial ceramic sealant (Huitian 2767). Synthetic air (20 vol.% O_2_ and 80 vol.% N_2_) was used at a rate of 150 mL/min on the feed side of the sample, while on the sweep side, 1 mL/min of Ne and 29 mL/min of He were used. The flow rates were regulated by mass flow controllers (EL-Flow^®^, Bronkhorst, AK Ruurlo, The Netherlands) in normal conditions (273.15 K, 101325 Pa). The concentration of the effluent was analyzed by an on-line gas chromatograph (Agilent 7890A) equipped with a Carboxen^®^ 1000 column (Merck, Darmstadt, Germany) and a thermal conductivity detector. Due to imperfect sealing, a small amount of N_2_ was also detected in the effluent and the leakage of oxygen was subtracted in the calculation of the oxygen permeation flux [37].

## 3. Results and Discussion

### 3.1. Phase Analysis of Powders

**Figure 1:**
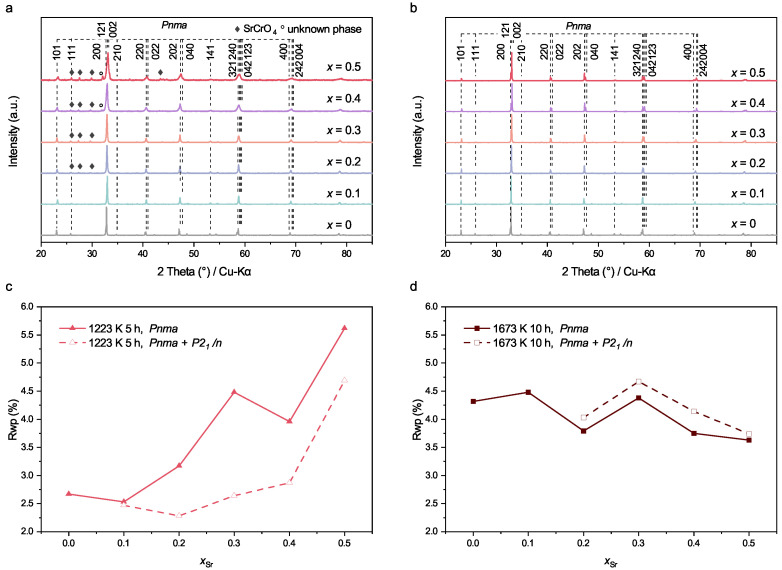
Room-temperature XRD patterns of Pr_1−x_Sr_*x*_(Cr,Mn,Fe,Co,Ni)O_3−δ_ (x= 0–0.5) powders calcined at (**a**) 1223 K and (**b**) 1673 K. Reflections of the main phase were indexed according to the results of Rietveld refinement using PrCrO_3_ (ICSD 251098) as the starting structure. The diamond symbols denote Bragg positions of SrCrO_4_ (ICSD 40922). Results of Rietveld refinements of corresponding powders calcined at 1223 K and 1673 K. The solid lines present the Rwp factors of refinements using PrCrO_3_ (Pnma, ICSD 251098) as the starting structure, while the dashed lines draw the Rwp factors of refinements using PrCrO_3_ (Pnma, ICSD 251098) and SrCrO_4_ ($P2_{1}/n$, ICSD 40922) as the starting structures.

The XRD patterns of Pr_1−x_Sr_*x*_(Cr,Mn,Fe,Co,Ni)O_3−δ_ (x= 0–0.5) powders calcined at 1223 K and 1673 K are shown in Figure 1a,b. Details of Rietveld refinement are listed in Table A3. After calcinating at 1223 K, all powders have an orthorhombic perovskite structure (Pnma). The reflections of SrCrO_4_ become noticeable along with an increasing *x*. For the powder with x=0.1, the solely visible reflection of SrCrO_4_ overlaps with the 111 reflection of the perovskite. Nevertheless, as shown in Figure 1c, a comparison between one-phase Rietveld refinement (PrCrO_3_ as starting structure) and two-phase Rietveld refinement (PrCrO_3_ and SrCrO_4_ as starting structures) leads to the conclusion that the powder with x=0.1 also has SrCrO_4_ as a secondary phase. This finding, although in good agreement with the work of Dąbrowa et al. [29], seems to be somehow contrary to the commonly understood high-entropy effect, namely that a single-phase solid solution tends to be formed when $\Delta S_{\mathrm{mix}}$ higher than 1.5R [2,4,38]. Note that $\Delta S_{\mathrm{mix}}$ increases as the *x* value increases from 0 to 0.5 (see Table A2).

In view of the Goldschmidt tolerance factor *t*, mixed entropy $\Delta S_{\mathrm{mix}}$, size difference of A-site cations $\Delta (R_{A})$, and size difference of B-site cations $\Delta (R_{B})$, the powders with *x* from 0 to 0.5 should be pure phase, as shown in Table A2:

- All the *t* factors are greater than 0.75 and become closer to 1 when *x* approaches 0.5, suggesting that a stable perovskite structure can be obtained [39].
- The $\Delta S_{\mathrm{mix}}$ increases from 1.61R for x=0 to 2.30R for x=0.5 as calculated by using Equation (2). Larger mixed entropy should indicate a more stable structure and thus pure phase upon substitution [2,4,38].
- The $\Delta (R_{A})$ and $\Delta (R_{B})$ are smaller than 6.5%, which means it is possible to form single-phase high-entropy perovskite [8,13,40].

Note that although $\Delta (R_{A})$ is smaller than 6.5%, it does increase along with the greater *x* due to the different ionic radii of Pr^3+^ and Sr^2+^ (see Table A1). The formation of the secondary phase may be correlated to the relatively large $\Delta (R_{A})$.

**Figure A1:**
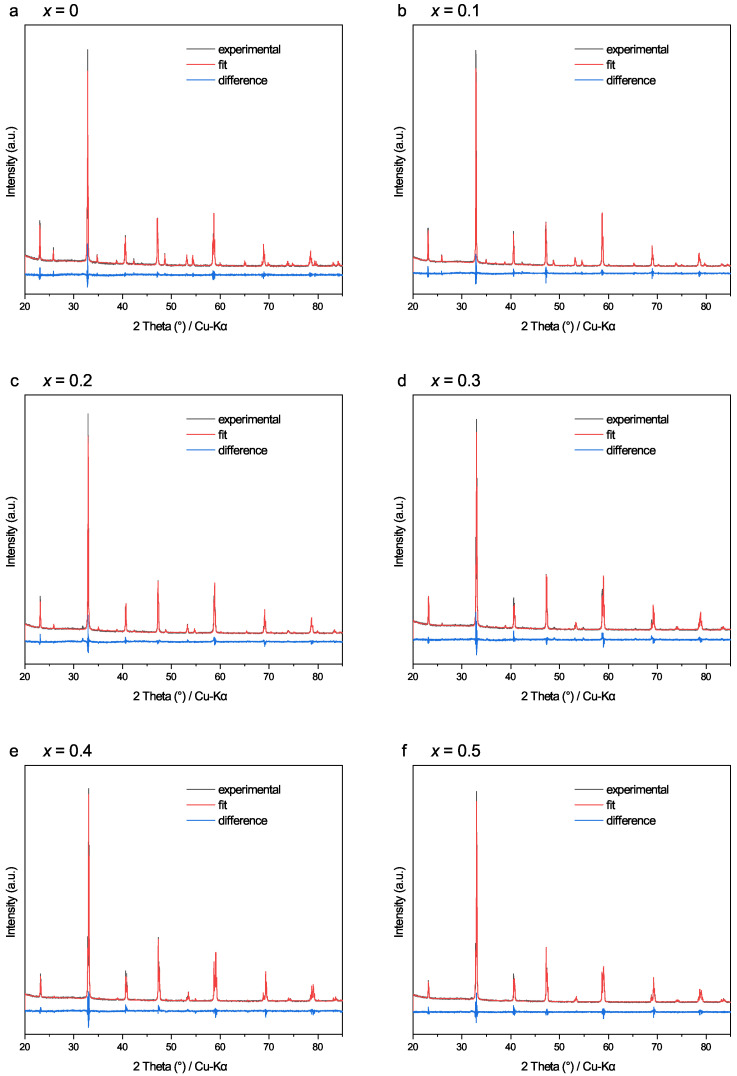
Rietveld refinements of Pr_1−x_Sr_*x*_(Cr,Mn,Fe,Co,Ni)O_3−δ_ (x= 0–0.5) powders treated at 1673 K. PrCrO_3_ (Pnma, ICSD 251098) was used as starting structure since the powders are pure phase after heating at 1673 K, as discussed in Section 3.1 in the main text.

However, when a higher calcination temperature of 1673 K is used, the powders are still pure phase even with a large amount of Sr (i.e., x=0.5), as proven by the XRD patterns in Figure 1b and the corresponding Rietveld refinements in Figure 1d and Figure A1. As shown in Table A3, the powders are considered as pure phase when treated after 1223 K for x=0, 1423 K for x∈[0,0.1], and 1673 K for x∈[0,0.5]. For the calcination temperature of 1223 K, only the sample with x=0 has Rwp and GOF factors from one-phase refinements that are smaller than those from two-phase refinements. For the calcination temperature of 1423 K and x∈[0,0.1], the Rwp and GOF factors from one-phase refinements are smaller than those from two-phase refinements. For the calcination temperature of 1673 K, all the Rwp and GOF factors from one-phase refinements are smaller than those from two-phase refinements. It seems that a high temperature is beneficial to maintain the perovskite structure and thus increase the strontium solubility. A higher temperature results in greater $T\Delta S_{\mathrm{mix}}$, and thus a more negative $\Delta G_{\mathrm{mix}}$ according to Equation (1), which can compensate the effect of large $\Delta (R_{A})$ and ensure the formation of the pure phase.

**Figure 2:**
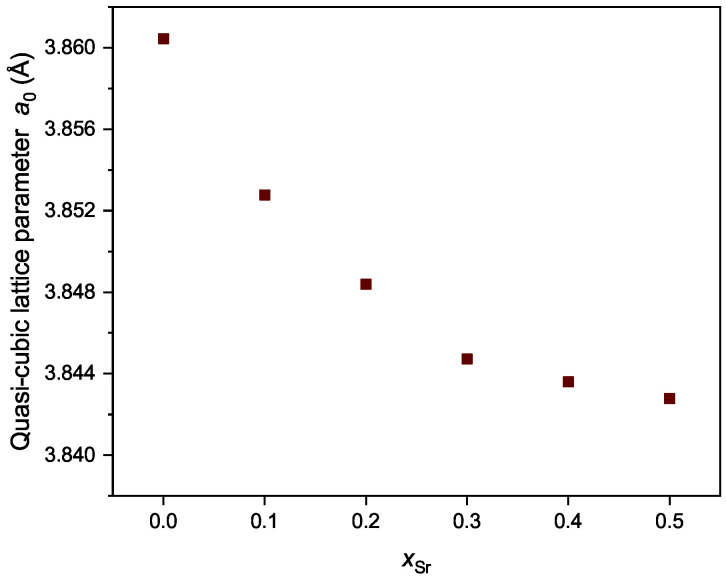
Quasi-cubic lattice parameter of Pr_1−x_Sr_*x*_(Cr,Mn,Fe,Co,Ni)O_3−δ_ (x= 0–0.5) powders after heating at 1673 K.

Since all the powders are pure phase and have an orthorhombic Pnma structure, the quasi-cubic lattice parameter $a_{0}$, which is calculated assuming the unit cell of the orthorhombic system is four times larger than the corresponding cubic system (i.e., $a\times b\times c=\sqrt{2}a_{0}\times 2a_{0}\times \sqrt{2}a_{0}$), is used to probe the influence of Sr content. The lattice parameters of the orthorhombic system can be found in Table A3. A sharp decrease in $a_{0}$ can be clearly seen from x=0 to x=0.3 (Figure 2), while this trend slows down for x=0.4 and x=0.5. The shrinkage of $a_{0}$ along with increasing Sr content, i.e., partial substitution of Pr^3+^ by Sr^2+^, indicates that charge compensation mechanism contributes more upon substitution, rather than the formation of oxygen vacancy [21]. Additionally, with more Sr in the composition, i.e., x∈[0.4,0.5], the impact of oxygen vacancy growths and thus endows the unit cell the ability to expand, so that the decreasing trend of the lattice parameters becomes slower. The relationship between $a_{0}$ and *x* gives a hint that Sr enters the unit cell of Pr_1−x_Sr_*x*_(Cr,Mn,Fe,Co,Ni)O_3−δ_, rather than evaporating during heating treatment. A more precise analysis is presented in Section 3.2. The influence of Sr content on the electrical conductivity and oxygen permeability will be discussed in Section 3.3 and Section 3.4.

**Figure 3:**
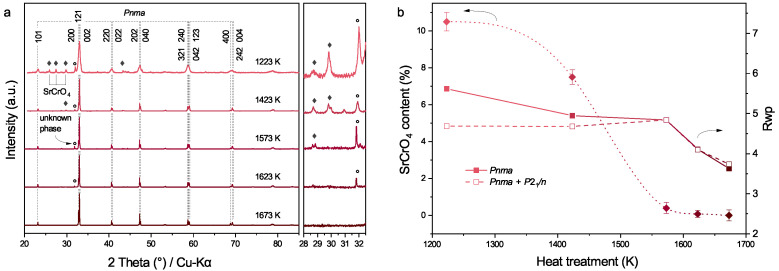
(**a**) Room-temperature XRD patterns of Pr_0.5_Sr_0.5_(Cr,Mn,Fe,Co,Ni)O_3−δ_ powders treated at temperatures in the range of 1223 K to 1673 K followed by natural cooling. The cooling rates are shown in Figure A2. Reflections of the main phase were indexed according to the Rietveld refinement using PrCrO_3_ (Pnma, ICSD 251098) as the starting structure. The diamond symbols denote Bragg positions of SrCrO_4_ ($P2_{1}/n$, ICSD 40922). (**b**) The influence of heating temperatures on the SrCrO_4_ content and the corresponding Rwp factors obtained by Rietveld refinements.

**Figure A2:**
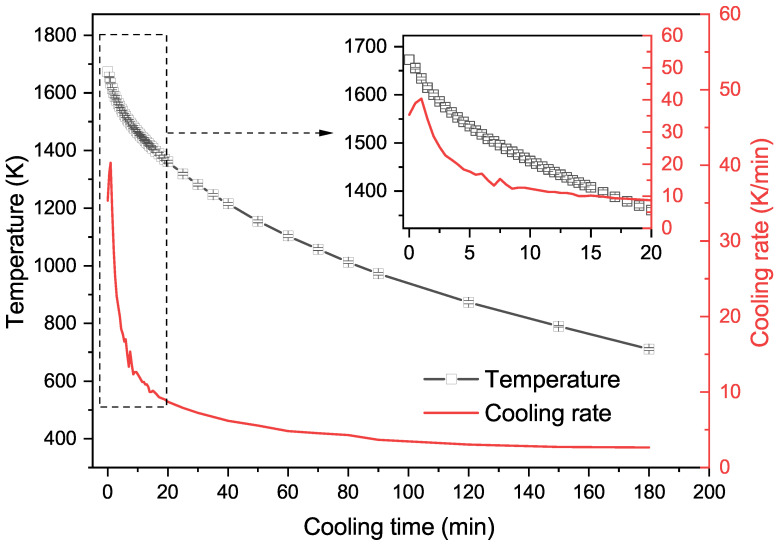
Temperature profile of the Carbolite HTF 1700 oven during natural cooling. The cooling rate is derived from the first-order derivative of the temperature curve. Ambient temperature: 297 ± 1 K. The cooling process was conducted three times. Error bars are inside the hollow squares.

To further investigate the influence of heating temperatures on the Sr solubility, uncalcined Pr_0.5_Sr_0.5_(Cr,Mn,Fe,Co,Ni)O_3−δ_ were divided into five portions and treated at temperatures from 1223 K to 1673 K followed by natural cooling. The results are visualized in Figure 3 and the cooling rate can be found in Figure A2. After cooling down to room temperature from 1223 K, reflections from SrCrO_4_ and an unknown phase are present between the 101 and 200 reflections of the perovskite phase (Figure 3a). The reflections of SrCrO_4_ and the unknown phase disappear after heating at 1623 K. The Rwp values of the Rietveld refinements in Figure 3b also indicate that after heating at 1673 K, one-phase refinement (Pnma) fits better than two-phase refinement ($Pnma+P2_{1}/n$). The content of SrCrO_4_ in powders decreases from 10.5% to 0% (under the detection limit of XRD) as the heating temperature varies from 1223 K to 1673 K.

**Figure A3:**
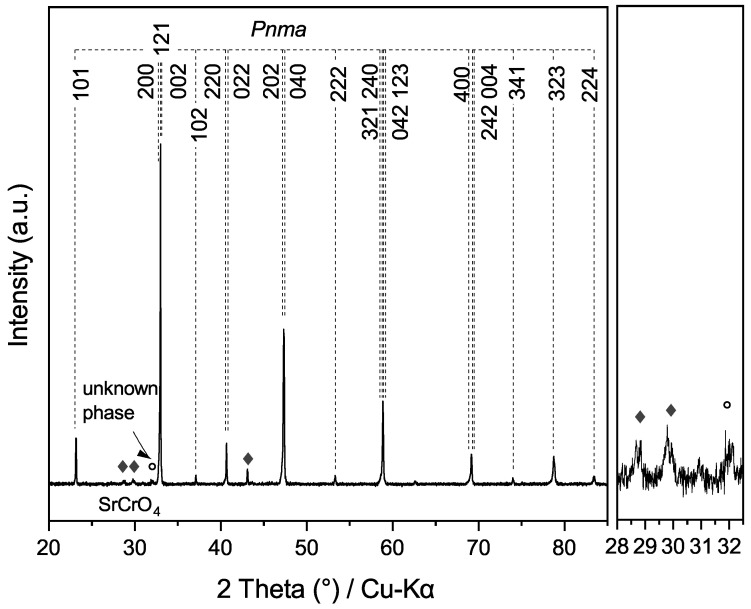
XRD pattern of Pr_0.3_Sr_0.7_(Cr,Mn,Fe,Co,Ni)O_3−δ_ powder treated after 1673 K for 10 h. Reflections were indexed according to the results of Rietveld refinement using PrCrO_3_ (ICSD 251098) as the starting structure.

Interestingly, increasing the treatment temperature to 1673 K did not bring about a pure phase for the Sr amount of x=0.7, as shown by the XRD pattern in Figure A3. Moreover, the surface and cross-section of the x=0.7 membranes were porous after sintering (Figure A6), which is not qualified as oxygen separation membranes. According to Equation (2), the mixed entropy $\Delta S_{\mathrm{mix}}$ increases with *x* from 0 to 0.5 and decreases with *x* from 0.5 to 1. Since Sr^2+^ has the largest ionic radius among Pr_1−x_Sr_*x*_(Cr,Mn,Fe,Co,Ni)O_3−δ_ and it reacts readily with Cr [21], we speculate that during the sintering process (1673 K for 10 h), Pr_0.3_Sr_0.7_(Cr,Mn,Fe,Co,Ni)O_3−δ_ is not stable and tends to release Sr to maximize $\Delta S_{\mathrm{mix}}$. In other words, when the doping ratio of Sr exceeds 0.5, the system has a tendency to decrease the amount of Sr, letting the amount of Sr equals the amount of Pr; the released Sr may react with Cr, forming SrCrO_4_.

### 3.2. Characterization of Membranes

**Figure 4:**
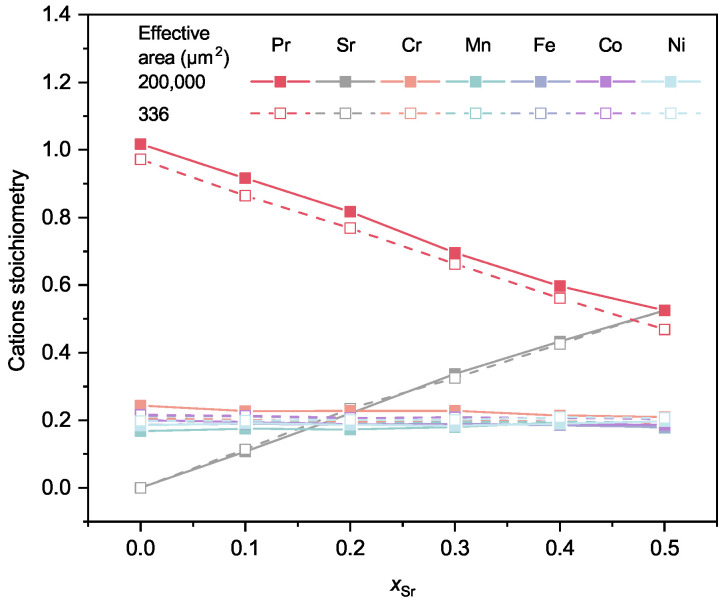
Cation stoichiometry of Pr_1−x_Sr_*x*_(Cr,Mn,Fe,Co,Ni)O_3−δ_ (x= 0–0.5) calculated from results of EDXS (Table A4 and Table A5) under the assumption that the sum of A-site cations and B-site cations is two. The solid lines present data obtained on an effective area of 200,000 µm^2^ by a lithium-drifted silicon detector while the dashed lines are data on an effective area of 336 µm^2^ by silicon drift detectors. The cross-sections of samples were vibratory-polished and sputtered with a carbon layer before measurement.

In Section 3.1, we have proved that the pure phase of Pr_1−x_Sr_*x*_(Cr,Mn,Fe,Co,Ni)O_3−δ_ with *x* ranging from 0 to 0.5 can be obtained after heating at 1673 K. The powders were then tableted and sintered at 1673 K for 10 h to prepare membranes. The vibratory-polished cross-sections of membranes were investigated by EDXS and SEM to gain insights into the content and distribution of elements in addition to the microstructure of membranes. The average compositions of the membranes are listed in Table A4 and Table A5, while the stoichiometry of the cations is illustrated in Figure 4. It is quite interesting that for both measurement areas of 200,000 µm^2^ and 336 µm^2^ the calculated stoichiometry matches well with the desired chemical formulae. The sum of Pr and Sr is about 1 and the other cations are all circling around 0.2. This finding suggests that during the calcination and sintering processes, there is no loss of elements, at least not of a single element.

**Figure 5:**
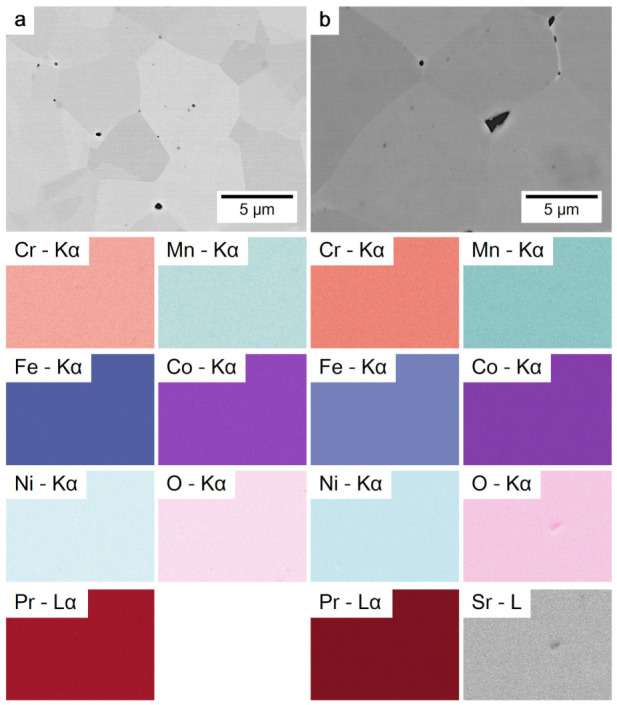
Electron channeling contrast images of vibratory-polished cross-sections of membranes (**a**) Pr(Cr,Mn,Fe,Co,Ni)O_3−δ_ and (**b**) Pr_0.5_Sr_0.5_(Cr,Mn,Fe,Co,Ni)O_3−δ_ and corresponding elemental distributions derived from silicon drift detectors.

The SEM investigation in the backscattered electron channeling contrast mode produced similar images of membranes with x=0 and x=0.5 (Figure 5a,b), indicating that no intergrowths are formed after introducing Sr into Pr_0.5_Sr_0.5_(Cr,Mn,Fe,Co,Ni)O_3−δ_. The electron channeling contrast comes from the change of the angle between the crystal lattice orientation and the incident electron beam. The following images further visualize the uniform distribution of each element in both samples without enrichment of Sr or Cr, which is found in Ln_1−x_Sr_*x*_(Cr,Mn,Fe,Co,Ni)O_3−δ_ (Ln = La, Pr, Nd, Sm, Gd), as reported in reference [21,29].

**Figure A4:**
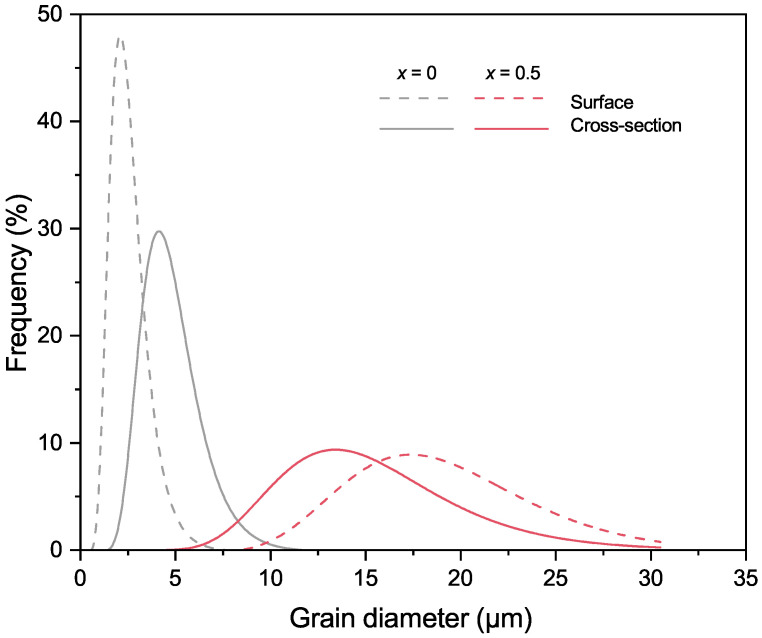
Grain size distribution of Pr_1−x_Sr_*x*_(Cr,Mn,Fe,Co,Ni)O_3−δ_ membranes (x=0,0.5) fitted with a log-normal density function. The dashed lines show the distribution of grains from the membrane surface, whereas the solid lines demonstrate the distribution of grains from the membrane cross-section. The grain diameter *d* is calculated according to $d=\sqrt{4A/\pi }$, where *A* is the measured area of grains. In each case, 100 grains were evaluated.

**Figure A5:**
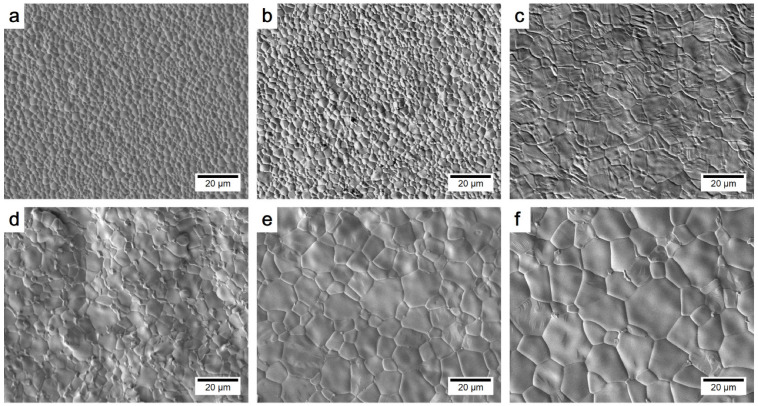
SEM images of the Pr_1−x_Sr_*x*_(Cr,Mn,Fe,Co,Ni)O_3−δ_ (x= 0–0.5) membrane surface. (**a**) x=0; (**b**) x=0.1; (**c**) x=0.2; (**d**) x=0.3; (**e**) x=0.4; (**f**) x=0.5. The close contact among the grains is responsible for enhanced electrical conductivity compared to La_1−x_Sr_*x*_(Cr,Mn,Fe,Co,Ni)O_3−δ_ (x= 0 – 0.3), as reported by Dąbrowa et al. [21].

**Figure A6:**
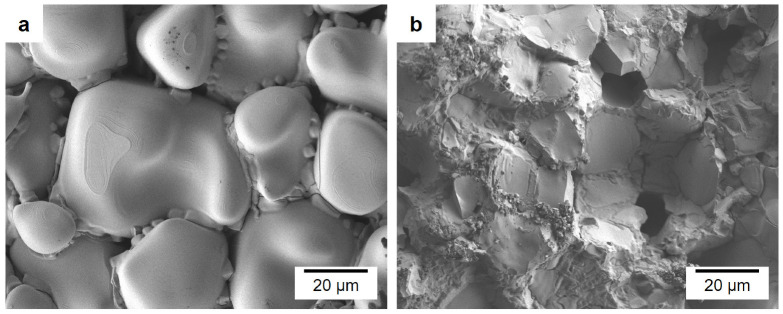
SEM images of Pr_0.3_Sr_0.7_(Cr,Mn,Fe,Co,Ni)O_3−δ_ membrane: (**a**) surface; (**b**) cross-section.

Figure 5 also demonstrates that the Sr content affects grain size of Pr_1−x_Sr_*x*_(Cr,Mn,Fe,Co,Ni)O_3−δ_ membranes. The average grain size increases with increasing strontium content, and is 4.6 µm^2^ for x=0 and 14.7 µm^2^ for x=0.5 (Figure A4). The Sr effect on grain size is explicitly shown by the SEM images of the membrane surface in Figure A5. A similar effect has been found in its low-entropy counterparts, e.g., La_1−x_Sr_*x*_Co_0.2_F_0.8_O_3_ [27,41,42]. Possible reasons could be the formation of a transient liquid phase or defects that facilitate mass transport during the sintering process [27]. Although this phenomenon is interesting and should be further investigated, it is beyond the scope of this work.

### 3.3. Electrical Conductivity

**Figure 6:**
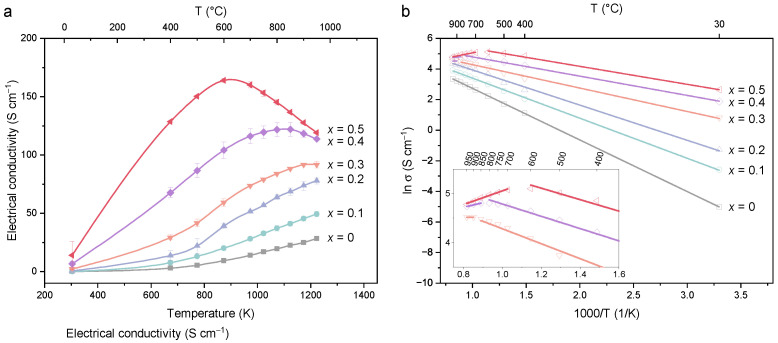
(**a**) Temperature-dependent electrical conductivity of Pr_1−x_Sr_*x*_(Cr,Mn,Fe,Co,Ni)O_3−δ_ (x= 0–0.5) membranes and (**b**) its Arrhenius plot.

The temperature dependence of the electrical conductivity of sintered samples was recorded in ambient air. Since the ionic conductivity is much smaller than the electronic conductivity in perovskite [25,26], the electrical conductivity can be regarded as the electronic conductivity. The conductivity increases with higher temperatures, reaches a maximum at a certain temperature, and then starts to decrease in the case of x>0.3, as depicted in Figure 6a, showing a transition from a semi-conductive to metallic behavior.

The semi-conducting behavior is related to a p-type small polaron hopping mechanism, i.e., the mobility of localized electronic carriers is thermally activated, while the decrease in conductivity after $T_{\max}$ (1073 K for x=0.4, 873 K for x=0.5) could be attributed to the loss of oxygen from the lattice [26,33]. With the loss of oxygen, the concentration of charge carriers is reduced as described by Equation (3) [26]:

(3) $$2\;B_{B}^{•}+O_{O}^{\times }↔2\;B_{B}^{\times }+V_{O}^{••}+\frac{1}{2}O_{2}$$

where $B_{B}^{•}$ and $V_{O}^{••}$ are tetravalent cations (electron holes) on the B-site and oxygen vacancy, respectively. Moreover, the overlap between the 3*d*-orbitals of the B-site cations and the 2*p*-orbitals of oxygen is decreased with the loss of oxygen, and consequently causing a decline in conductivity since the overlap is responsible for the electron transportation [43].

Table 1 lists the maximum conductivity, the corresponding temperature, as well as the activation energy determined from the linear part of the Arrhenius plot (Figure 6b). While the activation energies of Pr_1−x_Sr_*x*_(Cr,Mn,Fe,Co,Ni)O_3−δ_ (x= 0–0.5) are close to La_1−x_Sr_*x*_(Cr,Mn,Fe,Co,Ni)O_3−δ_ (x= 0–0.3), as reported by Dąbrowa et al. [21], the clear difference lies in the maximum values of electronic conductivity. We attribute the difference of higher electronic conductivity to the close contact among the grains as shown by the cross-sectional images in Figure 5a,b and the top-view images in Figure A5. High electronic conductivity is beneficial to applications involving solid oxide fuel cells or mixed ionic–electronic conductors [44]. It is obvious that adding Sr to the A-site significantly enhances the electronic conductivity and reduces the activation energy, which is 578% in $\sigma_{\max}$ and 34% in $E_{a}$ when comparing x=0.5 to x=0. Similar effects of Sr doping are also found in Sr-doped La, Pr, and Nd perovskites [26,33,34,45,46].

### 3.4. Oxygen Permeation

**Figure 7:**
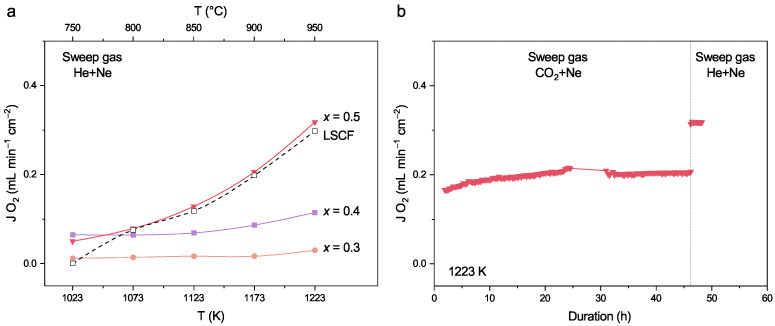
Oxygen flux of Pr_1−x_Sr_*x*_(Cr,Mn,Fe,Co,Ni)O_3−δ_ (x= 0–0.5) membranes as functions of (**a**) temperature and (**b**) duration. Test conditions: 150 mLmin^−1^ synthetic air as the feed gas, 29 mLmin^−1^ He or CO_2_ as the sweep gas, and 1 mLmin^−1^ Ne as the internal standard gas. Membrane thickness: 1.0 mm. Data for the La_0.6_Sr_0.4_Co_0.5_Fe_0.5_O_3−δ_ (LSCF) membrane were taken from our previous study [47].

The permeation performance was evaluated on Pr_1−x_Sr_*x*_(Cr,Mn,Fe,Co,Ni)O_3−δ_ (x= 0–0.5) membranes by using helium as sweep gas between the temperature of 1023 K to 1223 K. The oxygen fluxes of samples with x∈[0,0.2] are below the detection limit of gas chromatography and thus not shown here. Data of samples with x∈[0.3,0.5] are presented in Figure 7a. The influence of temperature and Sr content is evident: oxygen fluxes increase with elevated temperature and higher Sr content. Meanwhile, the magnitude of the increase in oxygen flux is also larger when *x* changes from 0.4 to 0.5 than when *x* changes from 0.3 to 0.4. Taking the changes in quasi-cubic lattice parameter (Figure 2) and electrical conductivity (Figure 6) into account, we can conclude that when increasing *x* from 0.4 to 0.5, more oxygen vacancies are introduced than increasing *x* from 0.3 to 0.4. The oxygen vacancy leads to expansion in cell volume, thus compensating for the effect of the tetravalent B-site cations and slowing down the decreasing trend of lattice parameter at high *x* values (Figure 2). Since the creation of one oxygen vacancy annihilates two electron holes (see Equation (3)), the $T_{\max}$ lowers with an increased *x*, and the decrease in electronic conductivity after $T_{\max}$ becomes steeper (Figure 6a).

The oxygen flux of membrane with *x* = 0.5 is close to that of La_0.6_Sr_0.4_Co_0.5_Fe_0.5_O_3−δ_ [47]. Moreover, the membrane was stable by using (almost) pure CO_2_ as sweep gas and the oxygen flux was not impaired, at least in 46.2 h (Figure 7b), showing good tolerance to CO_2_. After switching back to He as sweep gas, the flux returned from 0.21 mLmin^−1^cm^−2^ to 0.32 mLmin^−1^cm^−2^. Hence, the reduction of oxygen flux (ca. 34%) upon switching from He to CO_2_ is possibly due to the adsorbed CO_2_ on membrane surface as observed by several groups [32,48,49].

**Figure 8:**
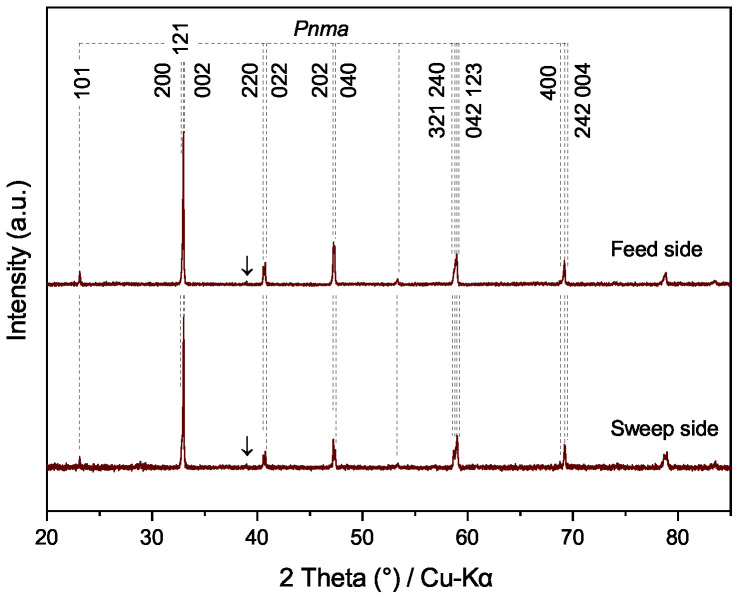
XRD patterns of the spent Pr_0.5_Sr_0.5_(Cr,Mn,Fe,Co,Ni)O_3−δ_ membrane after the experiment displayed in Figure 7 (CO_2_ + Ne sweep at 1223 K for 46.2 h and He + Ne sweep for 2 h). Reflections were indexed according to the results of Rietveld refinement using PrCrO_3_ (ICSD 251098) as the starting structure. Arrows: Bragg positions of CaCO_3_ (ICSD 52151) from XRD sample holders.

**Figure A7:**
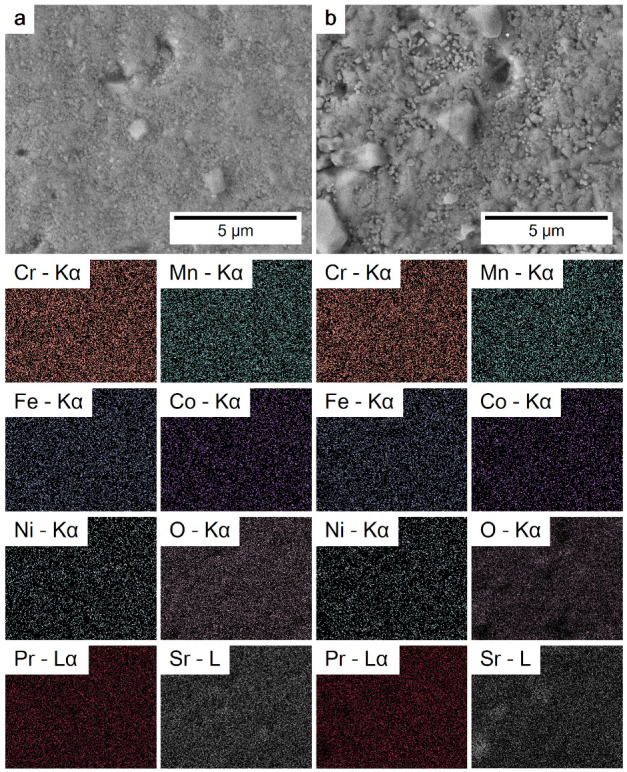
SEM images of the spent Pr_0.5_Sr_0.5_(Cr,Mn,Fe,Co,Ni)O_3−δ_ membrane and corresponding elemental distributions: (**a**) feed side and (**b**) sweep side. The surfaces were polished prior to the oxygen permeation measurement, and, therefore, no grain boundary is visible.

The phase structure and morphology of the spent Pr_0.5_Sr_0.5_(Cr,Mn,Fe,Co,Ni)O_3−δ_ membrane are shown in Figure 8 and Figure A7, respectively. On the feed and sweep sides, no secondary phase, such as SrCrO_4_, can be detected by XRD. Furthermore, no accumulation of Sr and Cr was found by EDXS analysis (Figure A7), all elements are uniformly distributed on both sides of the membrane surface after the oxygen permeation experiment of 48.2 h. The post-characterization of the spent membrane together with the long-term permeation test indicates that the high-entropy Pr_0.5_Sr_0.5_(Cr,Mn,Fe,Co,Ni)O_3−δ_ membrane possesses similar stability against CO_2_ as the low-entropy counterpart La_0.6_Sr_0.4_Co_0.5_Fe_0.5_O_3−δ_ [47].

## 4. Conclusions

Sr-doped high-entropy perovskites Pr_1−x_Sr_*x*_(Cr,Mn,Fe,Co,Ni)O_3−δ_ (x= 0–0.5) were successfully synthesized via a sol–gel method. Examination of phase purity by XRD, SEM, and EDXS demonstrated that raising the temperature of heating treatment can be used to increase the content of Sr in the pure phase. According to the results of Rietveld refinements, the quasi-cubic lattice parameter had a declined tendency towards higher Sr content and the tendency slowed down when x>0.3. Starting from this composition (x>0.3), a transition of semi-conductive to metallic behavior was observed in the electrical conductivity measurement within 1223 K. Furthermore, oxygen flux could be detected from 1023 K to 1223 K, and it was greatly enhanced by increasing the Sr content. The membrane Pr_0.5_Sr_0.5_(Cr,Mn,Fe,Co,Ni)O_3−δ_ exhibited a permeation behavior similar to La_0.6_Sr_0.4_Co_0.5_Fe_0.5_O_3−δ_, in view of the magnitude of oxygen flux and the chemical stability against CO_2_ in the range of tested temperatures.

## Figures and Tables

**Table 1:** Activation energies $E_{a}$ of Pr_1−x_Sr_*x*_(Cr,Mn,Fe,Co,Ni)O_3−δ_ (x= 0–0.5) membranes determined from the linear range of the Arrhenius plots shown in Figure 6b. $\sigma_{\max}$ and $T_{\max}$ are the maximum values of electrical conductivity and corresponding temperature among the measured data points.

*x*	$E_{a}$ (eV)	Temperature Range (K)	$\sigma_{\max}$ (S cm^−1^)	$T_{\max}$ (K)
0	0.29	303–1223	28.39	>1223
0.1	0.23	303–1223	49.37	>1223
0.2	0.20	303–1223	77.69	>1223
0.3	0.13	303–1153	81.82	>1153
0.4	0.11	303–1073	121.89	1073
0.5	0.10	303–873	163.99	873

## Data Availability

Not applicable.

## Abbreviations

The following abbreviations are used in this manuscript:

MIEC	mixed ionic–electronic conductor
BSCF	Ba_0.5_Sr_0.5_Co_0.8_Fe_0.2_O_3−δ_
XRD	X-ray diffraction
FE-SEM	field-emission scanning electron microscope
EDXS	energy-dispersive X-ray spectrometer
LSCF	La_0.6_Sr_0.4_Co_0.5_Fe_0.5_O_3−δ_

## Appendix A

**Table A1:** Ionic radii of elements in Pr_1−x_Sr_*x*_(Cr,Mn,Fe,Co,Ni)O_3−δ_ (x= 0–0.5) [50,51]. LS and HS stand for low spin and high spin, respectively.

Ion	Coordination Number	Ionic Radius (pm)
Pr^3+^	XII	132
Sr^2+^	XII	144
Cr^3+^	VI	61.5
Mn^3+^	VI	59^LS^, 64.5^HS^
Fe^3+^	VI	55^LS^, 64.5^HS^
Co^3+^	VI	54.5^LS^, 61^HS^
Ni^3+^	VI	56^LS^, 60^HS^
Cr^4+^	VI	55
Mn^4+^	VI	53
Fe^4+^	VI	58.5
Co^4+^	VI	53
Ni^4+^	VI	48
O^2-^	VI	140

**Table A2:** Goldschimdt tolerance factor *t*, size difference of A-site cations $\Delta (R_{A})$, size difference of B-site cations $\Delta (R_{B})$, and mixed entropy $\Delta S_{\mathrm{mix}}$ of Pr_1−x_Sr_*x*_(Cr,Mn,Fe,Co,Ni)O_3−δ_ (x= 0–0.5). LS and HS stand for low spin and high spin, respectively.

Sample	*t* (B^3+^)	*t* (B^4+^) *	Δ(R_A_) **	$\Delta (R_{B^{3+}})$	$\Delta (R_{B^{4+}})$ *	ΔS _**mix**_
x=0	0.98^LS^, 0.95^HS^	0.99	0.00%	4.47%^LS^, 2.99%^HS^	6.37%	1.61R
x=0.1	0.98^LS^, 0.95^HS^	1.00	2.70%	4.47%^LS^, 2.99%^HS^	6.37%	1.93R
x=0.2	0.98^LS^, 0.96^HS^	1.00	3.57%	4.47%^LS^, 2.99%^HS^	6.37%	2.11R
x=0.3	0.99^LS^, 0.96^HS^	1.01	4.06%	4.47%^LS^, 2.99%^HS^	6.37%	2.22R
x=0.4	0.99^LS^, 0.97^HS^	1.01	4.30%	4.47%^LS^, 2.99%^HS^	6.37%	2.28R
x=0.5	1.00^LS^, 0.97^HS^	1.02	4.35%	4.47%^LS^, 2.99%^HS^	6.37%	2.30R

* Assuming that the elements on B-site all have a valence of +4, which implies the absence of oxygen vacancies, ** The size difference is calculated as in Equation (A1).

(A1) $$\Delta \left(R_{A}\right)=\sqrt{\sum_{i=1}^{N}c_{i}\left(1-R_{A_{i}}/\left(\sum_{i=1}^{N}c_{i}R_{A_{i}}\right)\right)}$$

where $R_{A_{i}}$ is ionic radius of $i^{\mathrm{th}}$ cation on the A-site and $c_{i}$ is the mole fraction of $i^{\mathrm{th}}$ cation.

**Table A3:** Results of Rietveld refinements of Pr_1−x_Sr_*x*_(Cr,Mn,Fe,Co,Ni)O_3−δ_ (x= 0–0.5) after different heating treatments. As an initial point of the refinement, the PrCrO_3_ (Pnma, ICSD 251098) and SrCrO_4_ ($P2_{1}/n$, ICSD 40922) structures were used. The bold numbers highlight the smallest *x* value, from which the samples are not considered as pure phase (Pnma) any more, as reflected by their corresponding Rwp values. It is clear that with higher heating temperatures, the maximum *x* value in the pure phase of Pr_1−x_Sr_*x*_(Cr,Mn,Fe,Co,Ni)O_3−δ_ is increased.

	*x*	*a* (Å)	*b* (Å)	*c* (Å)	Rwp (%)	GOF	*P*2_1_/*n* (wt%)	*a* (Å)	*b* (Å)	*c* (Å)	Rwp (%)	GOF
		Pnma					$\mathrm{Pnma}+P2_{1}/n$					
1223 K 5 h	0	5.4698(2)	7.6982(3)	5.4407(2)	2.67	1.29	–	–	–	–	–	–
	**0.1**	5.4576(4)	7.7007(5)	5.4491(4)	**2.53**	1.23	1.8(2)	5.4577(4)	7.7008(5)	5.4491(4)	**2.47**	1.2
	0.2	5.4459(8)	7.6939(1)	5.4537(8)	3.17	1.5	7.7(2)	5.4461(6)	7.6939(8)	5.4536(6)	2.28	1.09
	0.3	5.4505(1)	7.6853(2)	5.4309(1)	4.48	2.3	11.3(2)	5.4501(7)	7.6862(1)	5.4305(7)	2.64	1.36
	0.4	5.423(2)	7.668(3)	5.453(2)	3.96	2.03	10.1(2)	5.4228(2)	7.668(2)	5.4531(2)	2.87	1.47
	0.5	5.434(6)	7.663(8)	5.470(6)	5.62	2.99	10.5(5)	5.433(5)	7.659(6)	5.466(5)	4.69	2.5
1423 K 10 h	0	5.4700(1)	7.6977(2)	5.4403(1)	3.71	1.71	–	–	–	–	–	–
	0.1	5.4522(1)	7.6999(2)	5.4526(1)	3.52	1.57	0.0(5)	5.4520(2)	7.6999(2)	5.4528(1)	3.52	1.57
	**0.2**	5.4379(4)	7.6940(6)	5.4552(4)	**3.48**	1.54	0.1(4)	5.4380(4)	7.6940(6)	5.4552(4)	**3.47**	1.54
	0.3	5.4578(4)	7.6842(5)	5.4284(4)	3.98	1.91	2.7(3)	5.4578(4)	7.6842(5)	5.4285(4)	3.93	1.89
	0.4	5.4204(4)	7.6700(7)	5.4568(5)	3.54	1.66	3.8(3)	5.4204(4)	7.6700(6)	5.4569(4)	3.43	1.61
	0.5	5.4166(7)	7.6589(1)	5.4597(7)	4.95	2.42	7.5(4)	5.4166(7)	7.6588(1)	5.4597(7)	4.68	2.29
1673 K 10 h	0	5.4774(3)	7.7098(4)	5.4496(3)	4.32	1.72	–	–	–	–	–	–
	0.1	5.4491(3)	7.7115(4)	5.4440(3)	4.48	2.21	–	–	–	–	–	–
	0.2	5.4354(3)	7.6912(4)	5.4535(3)	3.79	1.91	0.1(1)	5.4353(3)	7.6913(4)	5.4537(3)	4.03	2.03
	0.3	5.4583(3)	7.6788(4)	5.4239(3)	4.38	2.25	3.3(1)	5.4583(3)	7.6790(4)	5.4238(3)	4.67	2.40
	0.4	5.4656(2)	7.6657(3)	5.4211(2)	3.75	1.95	3.5(1)	5.4173(2)	7.6604(3)	5.4620(2)	4.14	2.16
	0.5	5.4623(2)	7.6628(3)	5.4232(2)	3.63	1.92	6.4(1)	5.4172(2)	7.6543(3)	5.4563(2)	3.74	1.97

**Table A4:** Average composition of Pr_1−x_Sr_*x*_(Cr,Mn,Fe,Co,Ni)O_3−δ_ (x= 0–0.5) obtained on an effective area of 200,000 µm^2^ by a lithium-drifted silicon detector.

Average Composition (at.%)									Ratio of Cations						
x	Pr	Sr	Cr	Mn	Fe	Co	Ni	O	Pr	Sr	Cr	Mn	Fe	Co	Ni
0	19.04	0.00	4.57	3.14	3.49	3.75	3.46	62.50	1.02	0.00	0.24	0.17	0.19	0.20	0.18
0.1	17.05	2.00	4.22	3.25	3.52	3.62	3.55	62.78	0.92	0.11	0.23	0.17	0.19	0.19	0.19
0.2	15.97	4.31	4.46	3.38	3.66	3.68	3.66	60.89	0.82	0.22	0.23	0.17	0.19	0.19	0.19
0.3	13.30	6.45	4.35	3.45	3.62	3.55	3.51	61.76	0.70	0.34	0.23	0.18	0.19	0.19	0.18
0.4	11.71	8.49	4.20	3.78	3.64	3.69	3.71	60.78	0.60	0.43	0.21	0.19	0.19	0.19	0.19
0.5	10.49	10.49	4.20	3.54	3.58	3.74	3.94	60.70	0.52	0.52	0.21	0.18	0.18	0.19	0.20

**Table A5:** Average composition of Pr_1−x_Sr_*x*_(Cr,Mn,Fe,Co,Ni)O_3−δ_ (x= 0–0.5) obtained on an effective area of 336 µm^2^ by silicon drift detectors.

Average Composition (at.%)									Ratio of Cations						
x	Pr	Sr	Cr	Mn	Fe	Co	Ni	O	Pr	Sr	Cr	Mn	Fe	Co	Ni
0	27.30	0.00	5.75	5.54	5.97	6.05	5.56	43.82	0.97	0.00	0.20	0.20	0.21	0.22	0.20
0.1	23.57	3.10	5.51	5.35	5.77	5.80	5.41	45.49	0.86	0.11	0.20	0.20	0.21	0.21	0.20
0.2	21.57	6.57	5.47	5.32	5.75	5.81	5.66	43.84	0.77	0.23	0.19	0.19	0.20	0.21	0.20
0.3	18.29	8.97	5.51	5.38	5.76	5.74	5.56	44.78	0.66	0.33	0.20	0.19	0.21	0.21	0.20
0.4	15.76	11.94	5.51	5.48	5.79	5.81	5.86	43.85	0.56	0.43	0.20	0.20	0.21	0.21	0.21
0.5	13.12	14.73	5.40	5.48	5.76	5.71	5.81	43.99	0.47	0.53	0.19	0.20	0.21	0.20	0.21
